# Commentary on: The Reverse Dual Plane: A Novel Technique for Endoscopic Transaxillary Breast Augmentation

**DOI:** 10.1093/asjof/ojae114

**Published:** 2024-11-22

**Authors:** Louis L Strock

See the Original Article here.

In their paper, “The Reverse Dual Plane: A Novel Technique for Endoscopic Transaxillary Breast Augmentation,” Ya et al describe a unique approach to the creation of a breast implant pocket. A traditional dual-plane approach allows for the creation of an implant pocket that leaves the central and upper implant pockets as subpectoral, and the lower pocket retroglandular. The authors, in effect, propose the opposite in their approach, with the upper approximately two-thirds of the implant pocket subfascial, and the lower third subpectoral. The report includes a total of 82 patients, all for primary breast augmentation, with 40 in the “Tebbetts dual-plane” group, and 42 in their “reverse dual-plane” group. The traditional dual-plane group had either dual Plane I or II pocket release. The reverse dual-plane technique is described as subfascial from the axilla to the fifth rib, where the plane is converted to subpectoral at the fifth rib. The inframammary fold (IMF) area was addressed by conversion to a more superficial plane from behind the pectoral muscle, which then results in a similar approach to the traditional dual plane relative to the soft tissue planes at the “new IMF.” Intercostal nerve blocks were performed in all patients. All implants used were Mentor Siltex, either shaped CPG or round, with a microtextured surface. Insertion sleeves were not used. Closed suction drains were used for 1 to 3 days, with IV antibiotics maintained for 2 days. Average follow-up was over 2 years in both patient groups.^1^

It is important to note that *microtextured implants* were used in all patients. This is important given that the reverse dual-plane pocket is mainly subfascial. Relative to their results, the authors report Baker III contracture in 1 patient in each group, with each requiring revision. Filiciani et al have shown that there is no significant difference in contracture rates, between dual-plane and subfascial implant approaches, as long as textured implants are used. That is compared with a reported *4-fold increase in contracture rates if smooth implants were used in a subfascial plane*.^2^ The authors of this study should be commended for following an evidence-based approach by the use of microtextured implants in all patients, which allows for a direct comparison of outcomes in capsular contracture to be based on an isolated comparison of the implant pocket only.

The authors’ technique is a modified transaxillary endoscopic approach. This is a technical issue separate from the type of pocket created. As an enthusiast for the transaxillary endoscopic approach, the thought process that I have followed has been to perform a technique for pocket creation, whether dual plane or subfascial, that is identical to that used with an inframammary approach, except for the equipment used. The endoscopic visualization allows for precise pocket creation and the ability to minimize blunt technique and optimize recovery.^3^ This can result in an enhanced recovery that minimizes pain and facilitates an early resumption of normal activity. It is important to note, however, that despite obvious technical expertise with the transaxillary endoscopic approach reflected in the authors’ experience, persistent issues with prolonged pain are central to their desire to move from a traditional dual-plane approach. Thus, the rationale and reasons for the development of the “reverse dual-plane” approach raise many questions. Pain control is usually not a challenge with transaxillary endoscopic breast augmentation in the dual plane, due to emphasis on the combination of precise tissue release, minimized use of blunt dissection, and the use of quick muscle relaxation agents intraoperatively, with oral muscle relaxants and anti-inflammatory medications in the early postoperative period.^4,5^ This protocol allows for dual-plane patients to experience a similar recovery with little difference compared with that seen with the subfascial approach, in my experience. There may be other important technical and medical differences that account for the ongoing challenges with pain control in this patient series. An important technical issue seen in the included video is that the authors divide the pectoralis muscle by prolonged contact with the instrument referred to as an electrotome, which may create a higher degree of heat injury to the muscle and surrounding tissues. This muscle division and tailored dual-plane creation can be accomplished with much less electrical instrument contact time if alternate cautery tools are used. Another important issue is that the tissue separation in dual-plane creation appears to be minimal in the review of video and may contribute to pain control challenges. These issues are at the core of breast augmentation technique, regardless of both incision choice (transaxillary or inframammary), and pocket location (dual plane, subfascial, or reverse dual plane).

The willingness to adopt a largely subfascial approach also suggests that the authors have not been satisfied with functional outcomes with their dual-plane approach. Issues with breast animation are most often cited as the reason for adopting the subfascial approach. Animation can be the result of a suboptimal dual-plane release, or one that is suboptimal in its medial extent, that can result in too much or too little muscle coverage of the medial implant area. This can and should be avoidable as an issue almost always. In this case, in addition to persistent issues with pain control, the authors also suggest that in their dual-plane patient group, there is a significant issue of what is described as “breast stiffness” or “firming,” especially with the arms abducted. They raise the issue in a section of the paper entitled “Breast Softness in a Different Position.” They report a significant decrease in this issue with their reverse dual-plane approach when compared with their dual-plane approach. Their data would suggest, however, that this remains a challenge, even with their reverse dual-plane approach, given that the issue persists in 23.8% of the reverse dual-plane group. The authors do not describe this issue in detail. The presentation of this issue raises some issues that merit additional consideration, given that a supine evaluation is less forgiving for evaluation of breast implant outcomes, likely due to tissue coverage of the implant device in a less favorable position when compared with the sitting position. It is my impression that this may represent a variant of breast animation, given that they note it is most prominent in the supine position with the arms abducted. As we move toward more patient-centric outcome analysis, such issues may increase in importance.

The approach to move to a subfascial pocket can work, as long as textured implants are used. The addition of the lower subpectoral pocket conversion in their approach can be viewed as unnecessary, given existing data on outcomes with a subfascial approach with textured implants. The use of insertion sleeves in their transaxillary approach is warranted, given exposure to the bacteria of hair follicles and sweat glands bacteria unique to this incision location. The conversion from subfascial to subpectoral at the fifth rib also merits careful consideration, especially for surgeons not highly experienced with endoscopic technique. The conversion to a deeper plane in this area can be met with the added risk of entering the chest cavity if endoscopic visualization is suboptimal, camera orientation is not carefully monitored, or if variations in ribcage anatomy make this pocket change hazardous if there are significant variations in rib projections in this area. Such anatomic variations are actually quite common. The need to then cut through the pectoral muscle to a superficial plane to modify the shape or level of the IMF, routinely necessary for many patients, raises the possible view that this is technically too complicated for many surgeons, especially those considering adding the transaxillary approach to their practices. These issues in combination suggest that the best approach may be to optimize dual-plane technique, especially if only smooth implants are used.

The authors are to be commended for their unique and innovative technique presented. There are many issues raised and lessons to be learned from the experience that the authors have presented. Transaxillary breast implant placement can now be performed with a choice between dual plane, subfascial, and now reverse dual-plane approaches, with excellent outcomes. As we now know, the pocket choice is linked to the implant to be used.^2^ The experience shown, in my view, also serves to emphasize the utility of the endoscope in allowing for creative approaches to the creation of a breast implant pocket using a transaxillary incision.
